# The P681H Mutation in the Spike Glycoprotein of the Alpha Variant of SARS-CoV-2 Escapes IFITM Restriction and Is Necessary for Type I Interferon Resistance

**DOI:** 10.1128/jvi.01250-22

**Published:** 2022-11-09

**Authors:** Maria Jose Lista, Helena Winstone, Harry D. Wilson, Adam Dyer, Suzanne Pickering, Rui Pedro Galao, Giuditta De Lorenzo, Vanessa M. Cowton, Wilhelm Furnon, Nicolas Suarez, Richard Orton, Massimo Palmarini, Arvind H. Patel, Luke Snell, Gaia Nebbia, Chad Swanson, Stuart J. D. Neil

**Affiliations:** a Department of Infectious Diseases, King’s College London, London, United Kingdom; b MRC-University of Glasgow Centre for Virus Research, Glasgow, United Kingdom; c Centre for Clinical Infection and Diagnostics Research, Department of Infectious Diseases, Guy’s and St Thomas’ NHS Foundation Trust, London, United Kingdom; d UKRI Genotype-2-Phenotype Consortium, London, United Kingdom; St. Jude Children's Research Hospital

**Keywords:** IFITM, SARS-CoV-2, type 1 interferon, VOC, furin cleavage site

## Abstract

The appearance of new dominant variants of concern (VOC) of severe acute respiratory syndrome coronavirus type 2 (SARS-CoV-2) threatens the global response to the coronavirus disease 2019 (COVID-19) pandemic. Of these, the alpha variant (also known as B.1.1.7), which appeared initially in the United Kingdom, became the dominant variant in much of Europe and North America in the first half of 2021. The spike (S) glycoprotein of alpha acquired seven mutations and two deletions compared to the ancestral virus, including the P681H mutation adjacent to the polybasic cleavage site, which has been suggested to enhance S cleavage. Here, we show that the alpha spike protein confers a level of resistance to beta interferon (IFN-β) in human lung epithelial cells. This correlates with resistance to an entry restriction mediated by interferon-induced transmembrane protein 2 (IFITM2) and a pronounced infection enhancement by IFITM3. Furthermore, the P681H mutation is essential for resistance to IFN-β and context-dependent resistance to IFITMs in the alpha S. P681H reduces dependence on endosomal cathepsins, consistent with enhanced cell surface entry. However, reversion of H681 does not reduce cleaved spike incorporation into particles, indicating that it exerts its effect on entry and IFN-β downstream of furin cleavage. Overall, we suggest that, in addition to adaptive immune escape, mutations associated with VOC may well also confer a replication and/or transmission advantage through adaptation to resist innate immune mechanisms.

**IMPORTANCE** Accumulating evidence suggests that variants of concern (VOC) of SARS-CoV-2 evolve to evade the human immune response, with much interest focused on mutations in the spike protein that escape from antibodies. However, resistance to the innate immune response is essential for efficient viral replication and transmission. Here, we show that the alpha (B.1.1.7) VOC of SARS-CoV-2 is substantially more resistant to type I interferons than the parental Wuhan-like virus. This correlates with resistance to the antiviral protein IFITM2 and enhancement by its paralogue IFITM3. The key determinant of this is a proline-to-histidine change at position 681 in S adjacent to the furin cleavage site, which in the context of the alpha spike modulates cell entry pathways of SARS-CoV-2. Reversion of the mutation is sufficient to restore interferon and IFITM2 sensitivity, highlighting the dynamic nature of the SARS CoV-2 as it adapts to both innate and adaptive immunity in the humans.

## INTRODUCTION

Both severe acute respiratory syndrome coronavirus type 1 (SARS-CoV-1) and SARS-CoV-2 enter target cells through the interaction of their S proteins with the angiotensin-converting enzyme 2 (ACE2) cell surface receptor. Upon attachment and uptake, the S glycoprotein trimer is cleaved by cellular proteases such as cathepsins and TMPRSS (transmembrane proteases serine subfamily) members at two positions—the S1/S2 junction and the S2′ site—to facilitate the activation of the fusion mechanism. Similar to more distantly related beta-CoVs, but so far unique in known sarbecoviruses, the SARS-CoV-2 glycoprotein contains a polybasic furin cleavage site (FCS) with a 681-PRRAR*S-685 sequence at the S1/S2 junction. This allows the S precursor to be additionally processed to the S1 and S2 subunits by furin-like proteases before viral release from the previously infected cell (1). This leads to a proportion of processed S being present on the virion before engagement with the target cell, allowing rapid activation and fusion at or near the cell surface by TMPRSS2. The importance of the FCS is highlighted by the observations that it enhances SARS-CoV-2 replication specifically in airway epithelial cells and that it is essential for efficient transmission in animal models (2).

The alpha variant of SARS-CoV-2 arose in the southeast of England in autumn 2020 and rapidly spread across the world in the first months of 2021. Various studies suggested that alpha had an increased transmissibility between individuals (3–5). Alpha contains nine amino acid residue changes in S, including a deletion of amino acid residues H and V in the N-terminal domain (NTD) at position 69/70 (thought to increase S incorporation into virions), a single amino acid deletion of Y144 (thought to assist NTD antibody neutralization escape), and an N501Y mutation in the receptor-binding domain (RBD), which enhances ACE2 binding affinity (6, 7). Together, these changes have been shown to reduce efficiency of neutralization by some antibodies (8), but compared to the later variants of concern (VOC) delta and omicron, it is not thought to be a major adaptive immune escape variant. Alpha also acquired a P681H change in the FCS, which has been proposed to increase the accessibility of the site by furin, leading to enhanced cleavage as well as more efficient cell-to-cell fusion and syncytium formation (9–12). Since early 2021, several other VOC have emerged with mutations in the FCS, including kappa, delta, and omicron (12, 13). Both kappa and delta contained the P681R mutation; however, only delta superseded alpha and became a globally dominant variant in the summer of 2021. In late 2021, the delta variant was in turn displaced by the omicron variant, which contains the P681H mutation in its FCS.

We and others have previously demonstrated that the ancestral SARS-CoV-2 is variably sensitive to entry inhibition by the interferon-regulated interferon-induced transmembrane protein (IFITM) family and that this can be modulated by the FCS (2, 14, 15). IFITM1, -2, and -3 are transmembrane proteins that exert antiviral activity against diverse enveloped viruses by blocking fusion of the viral and cellular membranes (16, 17). While IFITM1 localizes primarily to the plasma membrane, IFITM2 and IFITM3 are internalized via a conserved YxxΦ endocytic motif to occupy both distinct and overlapping endosomal compartments. However, it was demonstrated previously that the IFITM proteins can oligomerize with each other in heterologous complexes (18, 19). The sensitivity of a given virus to individual IFITM proteins is largely determined by its route of cellular entry. We showed previously that for a prototypic Wuhan-like SARS-CoV-2 isolate from early 2020, IFITM2 reduced viral entry and contributed to type I interferon (IFN-I)-induced inhibition in human cells (14). Sensitivity to IFITM2 could be markedly enhanced by deletion of the FCS, suggesting that furin processing ameliorated SARS-CoV-2 sensitivity to IFITM2 restriction at least to some extent. We therefore postulated that the altered cleavage site of VOC with mutations in the FCS may have consequences for their sensitivity to IFN-I and IFITMs. Here, we demonstrate that of the alpha, beta, gamma, kappa, delta, and omicron variants, only the S of the alpha variant is resistant to IFITM restriction in A549-ACE2-IFITM cells. We also demonstrate that the ΔCT (cytoplasmic tail) mutation commonly used in improving SARS-CoV-2 pseudotyped lentiviral vector (PLV) infectivity masks the IFITM resistance of alpha PLVs by conferring increased cathepsin dependence. Furthermore, we show that the alpha variant is resistant to IFN-β in both A549-ACE2 and Calu-3 cells, and this resistance can be abolished by reversion of the P681H mutation.

## RESULTS

### The S proteins of currently circulating variants display different sensitivities to IFITMs in A549-ACE2 cells.

**FIG 1:**
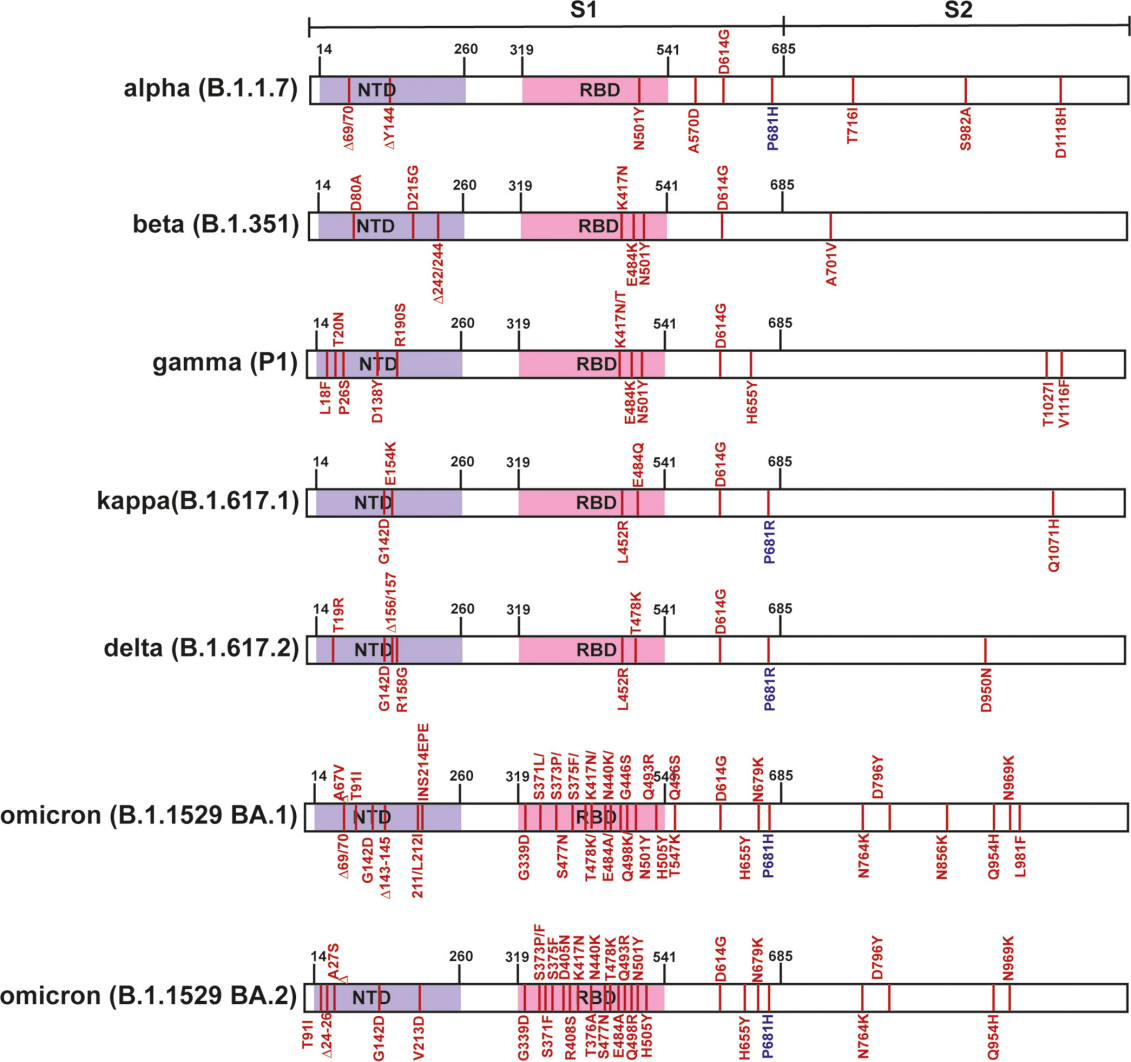
SARS-CoV-2 variants of concern spike sequences. Schematic of spike protein domains of the different variants of concern relative to the original Wuhan spike sequence: alpha, beta, gamma, delta, and omicron. The different mutations between the variants are represented in red.

**FIG 2:**
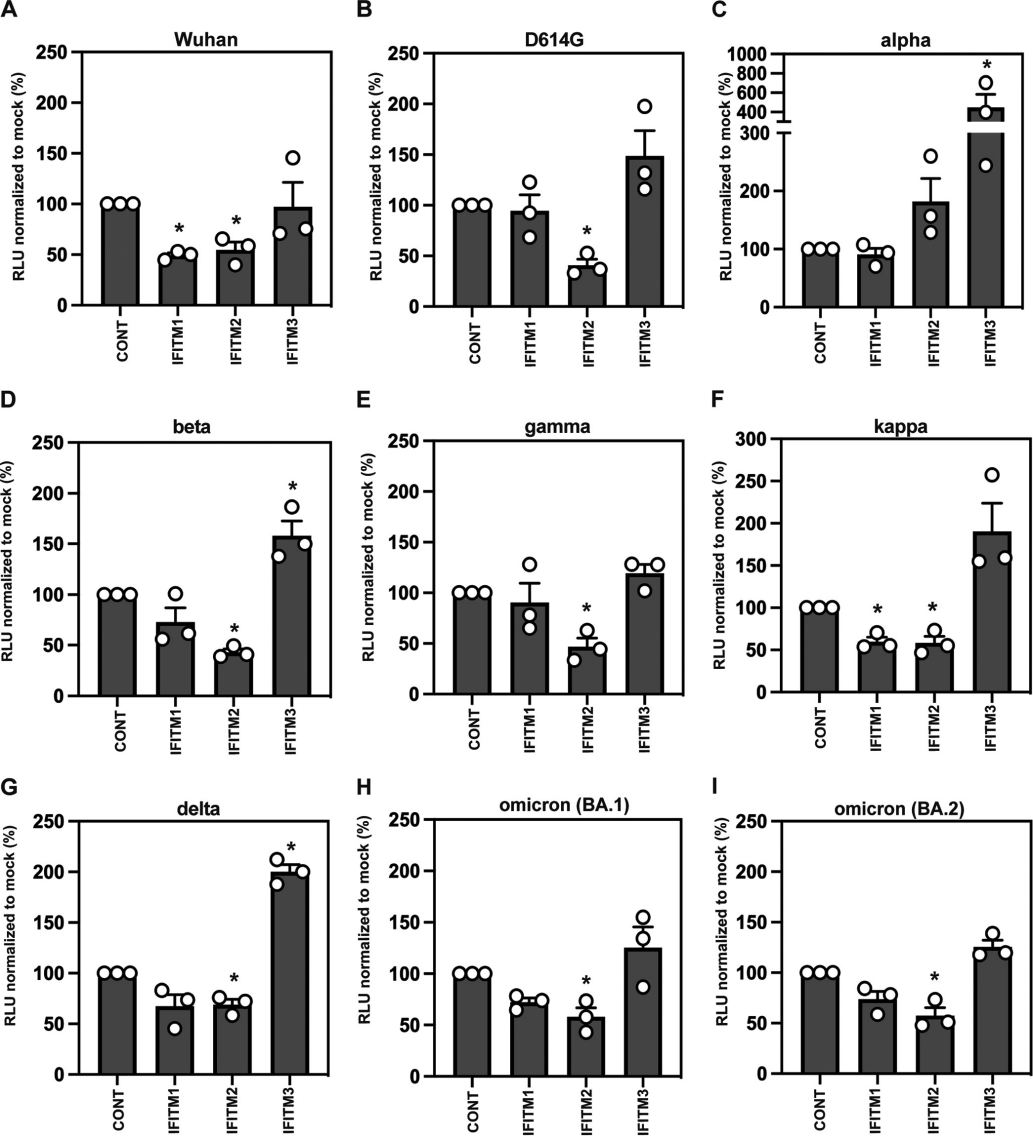
IFITM sensitivity of SARS-CoV-2 variants of concern. IFITM sensitivity of PLVs bearing full-length Wuhan, D614G, alpha, beta, gamma, kappa, delta, and omicron spike in A549-ACE2 cells stably expressing the individual IFITMs. PLV entry was quantified by luciferase activity 48 h after infection and normalized to control (CONT) cells. Data are means and standard errors of the means (SEM; *n* = 3). Statistics were calculated in Prism using analysis of variance (ANOVA). *, *P* = 0.05 relative to control values.

Over 2020 and 2021, several major VOC arose—alpha (B.1.1.7) in the United Kingdom, beta (B.1.351) in South Africa, gamma (P1) in Brazil, delta (B.1.617.2) in India, and most recently the omicron family (B.1.1.529) in South Africa (13). All of these variants have multiple changes in the S protein that could potentially affect the entry process (Fig. 1). Of particular interest, the alpha, delta and omicron variants contain mutations in the polybasic cleavage site which have been postulated to enhance S cleavage: P681H in alpha and omicron and P681R in delta (20–22). We therefore compared the sensitivity of PLVs bearing full-length, untruncated SARS-CoV-2 spike proteins of these VOC to entry inhibition the presence of IFITM proteins. As expected, all VOC PLVs produced were infectious on A549-ACE2 cells, although efficiency was variable (see Fig. S1A in the supplemental material). We then used these PLVs to infect A549-ACE2 cells stably expressing the individual IFITMs (Fig. S1B; Fig. 2A to I). The spike protein with the D614G mutation, which became dominant early in the first wave of the pandemic, displayed a sensitivity to IFITM2 similar to that of the previously characterized Wuhan-1 S but was resistant to both IFITM1 and IFITM3 (Fig. 2A and B) (14, 23). We then compared the IFITM sensitivities of alpha, beta, gamma, kappa, delta, and omicron (BA.1 and BA.2) as PLVs (Fig. 2C to I). The alpha S (Fig. 2C) appeared completely insensitive to IFITM1, -2, and -3, while beta, gamma, kappa, delta, and both omicron spikes retained some sensitivity to IFITMs 1 and/or 2. We noted that kappa and delta (Fig. 2F and G), which both contain the P681R mutation, retained some sensitivity to both IFITM1 and -2. Interestingly the alpha variant, and to some extent delta, also appeared to be significantly enhanced by IFITM3. Such enhancement by IFITMs has been previously documented in the human seasonal CoV OC43 and in SARS-CoV-2 under specific assay conditions when IFITM2 is knocked down postinfection in IFN-treated cells (24, 25). To confirm the enhancement we observed with alpha was due to IFITM3, we pretreated A549-ACE2-IFITM3 cells with cyclosporine H, a compound known to drive IFITM3 to ubiquitin-dependent degradation (9, 25, 26). We found that overnight treatment with CSH was able to reduce expression levels of all three IFITMs and led to specific abolishment of IFITM3 enhancement of alpha PLVs while having no effect on D614G PLVs (Fig. S1C to E).

### The ΔCT mutation increases PLV infectivity but confers greater cathepsin dependence and IFITM2 sensitivity to D614G and alpha PLVs.

**FIG 3:**
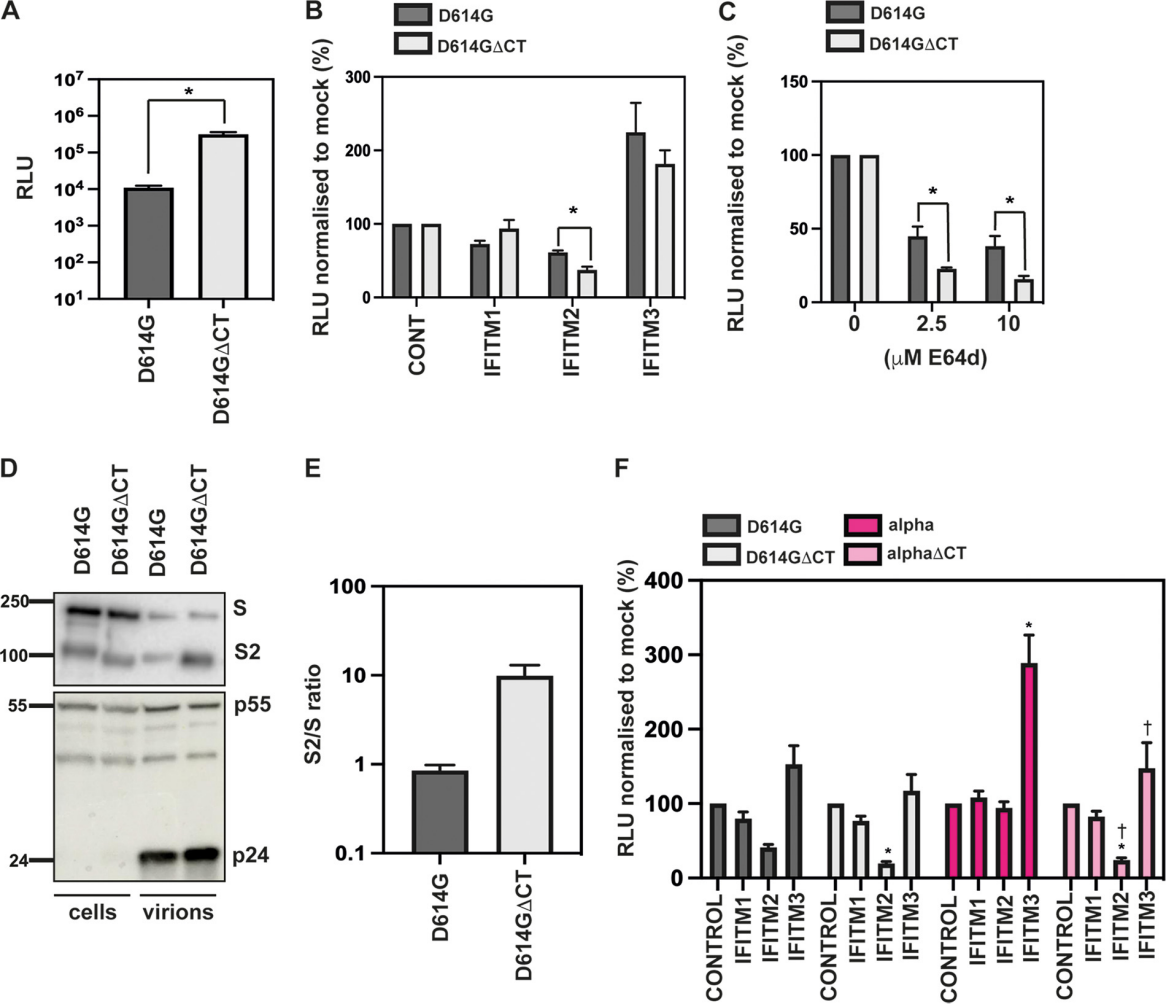
The ΔCT mutation in the D614G and alpha viruses confers IFITM2 sensitivity by increasing cathepsin dependence. (A) D614G or D614GΔCT PLVs were used to infect A549-ACE2 cells, and infectivity was measured by luciferase activity 48 h later. Raw relative light units (RLU) are shown. (B) D614G or D614GΔCT PLVs were used to infect A549-ACE2-IFITM cells, and infectivity was measured by luciferase activity 48 h later. Percent infection normalized to control values without IFITM is shown. (C) A549-ACE2 cells were pretreated with 2.5 μM or 10 μM E64d prior to infection with D614G or D614GΔCT PLVs for 48 h. Infection was measured by luciferase activity, and infection was normalized to mock-treated cells. (D) Representative immunoblot of cell lysates and supernatant from PLV production. Supernatant was purified through a 20% sucrose cushion for 1 h at 18,000 × *g* prior to lysis. (E) Quantification of the S2/S ratio in 3 independent immunoblots of the purified supernatant used for panel D. (F) D614G, ΔCT, alpha, or alpha ΔCT PLVs were used to infect A549-ACE2-IFITM cells for 48 h, and infection was quantified by luciferase activity. Infection is normalized to control values. Data are means and SEM (*n* = 3). Statistics were calculated in Prism using ANOVA. Asterisks and daggers indicate significance (*P* < 0.05) between control cells and cells expressing individual IFITMs and between different IFITM/drug conditions, respectively.

Deleting the last 19 amino acids of SARS-CoV-2 spike increases spike incorporation and infectivity of PLVs and is common practice among many groups studying SARS CoV-2 (27, 28). Truncation of the cytoplasmic tail results in the deletion of a suboptimal endoplasmic reticulum retention signal (ERRS) and increased accumulation of the spike at the surface, where it is incorporated into PLVs. However, the site of coronavirus assembly is not at the plasma membrane, and the spike goes through considerable posttranslational modifications in the ER-Golgi apparatus intermediate compartment (ERGIC) (29). To test whether deletion of the last 19 amino acids affected IFITM phenotypes, we generated a D614GΔCT mutant and tested infectivity in A549-ACE2 cells of these PLVs relative to the full-length D614G spike as PLVs (Fig. 3A). The ΔCT mutant exhibited a 28-fold boost in infectivity (Fig. 3A). However, the D614GΔCT PLVs were 2-fold more sensitive to IFITM2 (Fig. 3B). This was consistent with an increase in sensitivity of these PLVs to E64d, an inhibitor of cathepsins B/L at both 2.5 μM and 10 μM (Fig. 3C). Next, to confirm if there were phenotypic differences in the spike of D614GΔCT spikes during PLV production, D614G and D614GΔCT PLVs were immunoblotted for spike and Gag in both the cell lysates and purified supernatant of PLV production (Fig. 3D). Intriguingly, the D614GΔCT mutant showed a 10-fold increase in S1/S2 processing (Fig. 3E). Although increased spike processing was surprising given an increased dependence on cathepsins B and L, it could be that, although it was more processed, the D614GΔCT spike is in a conformation where the second cleavage site is less accessible, resulting in increased cathepsin dependence. Finally, to confirm whether the ΔCT mutation is sufficient to overcome the IFITM2 resistance observed with the alpha spike, alphaΔCT was generated and its IFITM sensitivity was tested (Fig. 3F). Strikingly, the ΔCT mutation rendered the previously resistant alpha spike highly sensitive to IFITM2. Additionally, the 3-fold enhancement we previously found with alpha in this system was abolished by the ΔCT mutation. Overall, these data suggest that the ERRS plays a significant role in the posttranslational modifications of spike, and in turn, this has consequences for the route of viral entry and sensitivity to antiviral proteins. Given the significant effect of this mutation on IFITM sensitivity and route of entry of D614G and alpha viruses, we advise caution in interpreting data of phenotypes involving differential viral entry utilizing ΔCT spikes.

### SARS-CoV-2 alpha variant is IFITM resistant.

**FIG 4:**
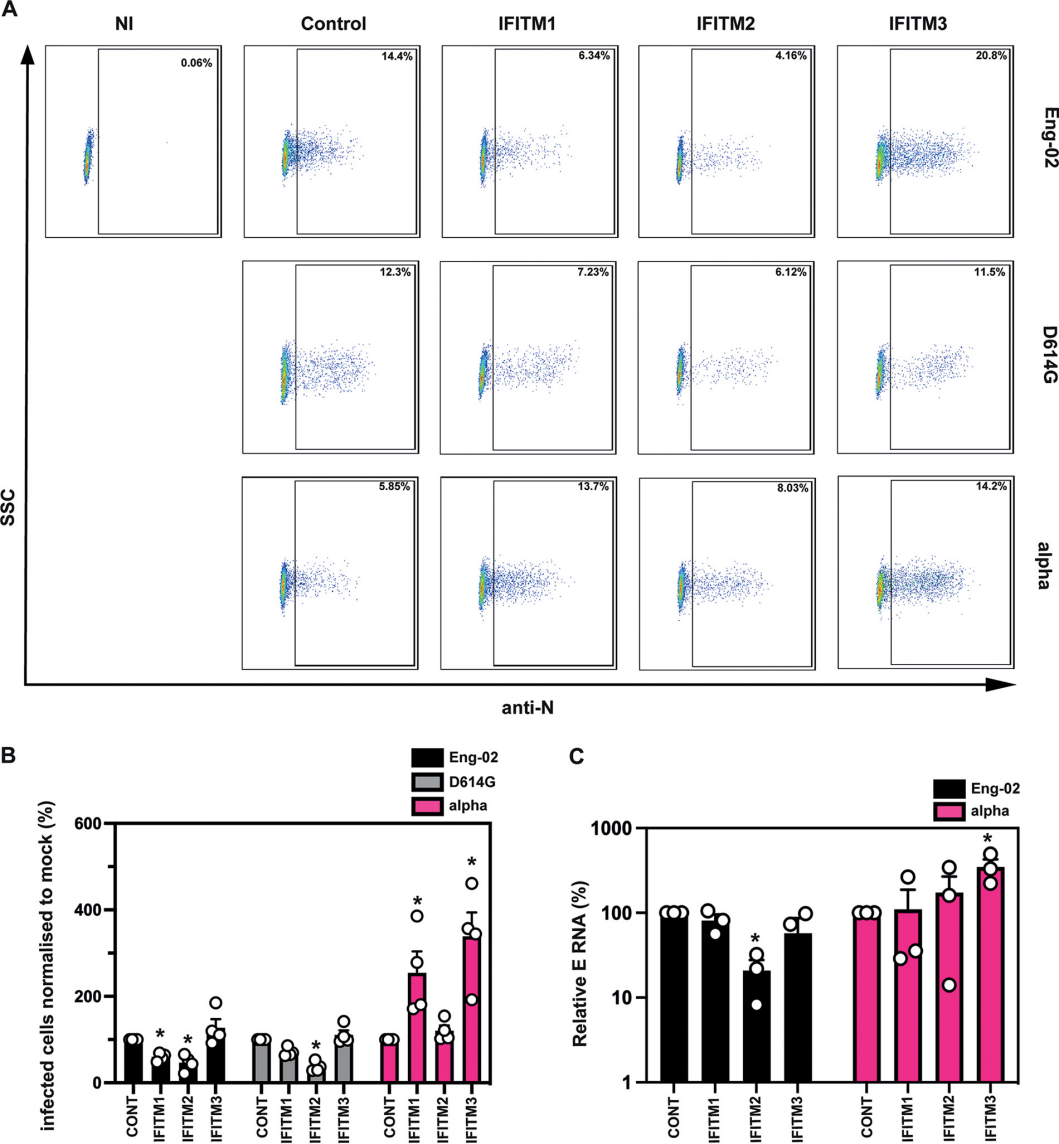
The alpha variant of SARS-CoV-2 is resistant to IFITMs. (A) Representative fluorescence-activated cell sorting plots of intracellular N staining of infected A549-ACE2-IFITM cells. NI, noninfected; SSC, side scatter. (B) Quantification of intracellular N staining by flow cytometry of A549-ACE2 IFITM cells infected with England-02, Wuhan D614G, and alpha. A549-ACE2 cells expressing the individual IFITMs were infected with England-02, D614G, or alpha isolates for 48 h. Infection was measured by determining the percentage of N-positive cells by flow cytometry. Data were analyzed in FlowJo. (C) Infection of A549-ACE2 stably expressing the individual IFITMs with England-02 and alpha viruses at an MOI of 0.01. Infection was quantified by RT-qPCR of E gene relative to GAPDH 48 h later; values are E mRNA levels relative to GAPDH. Data are means and SEM (*n* = 3). Statistics were calculated in Prism using ANOVA. Asterisks and daggers indicate significance (*P* < 0.05) between control cells and individual IFITMs and between different IFITM/drug conditions, respectively.

Next, we sought to confirm that the native alpha virus demonstrated a phenotype on our IFITM-expressing cells similar to that of the PLVs. We infected A549-ACE2 cells stably expressing the individual IFITMs with England-02, D614G, or alpha isolates and measured the percentage of N-positive cells by flow cytometry (Fig. 4A and B) and the level of intracellular E RNA by quantitative PCR (qPCR) (Fig. 4C) at 48 h postinfection. We found that England-02 and D614G isolates were IFITM2 sensitive, while alpha was insensitive to inhibitory effects of all three IFITMs. Also, we again noted significant enhancement of infection in the presence of IFITM3, consistent with our PLV experiments. However, while alpha PLV is weakly enhanced by IFITM2, the native virus was enhanced by IFITM1. We concluded that IFITM1 and -2 can enhance alpha infection, but in a variable manner that may be due to the ability of IFITMs to cycle through multiple cell compartments. Furthermore, both delta and omicron viruses displayed sensitivity to both IFITM2 and IFITM3 (Fig. S2). Thus, the alpha variant of SARS CoV-2, unique among the current VOC, is fully IFITM resistant in A549-ACE2s. Furthermore, the IFITM3 enhancement of alpha infection is reproducible between PLVs and native virus.

### The alpha variant is less sensitive to IFN-β than an early pandemic isolate.

**FIG 5:**
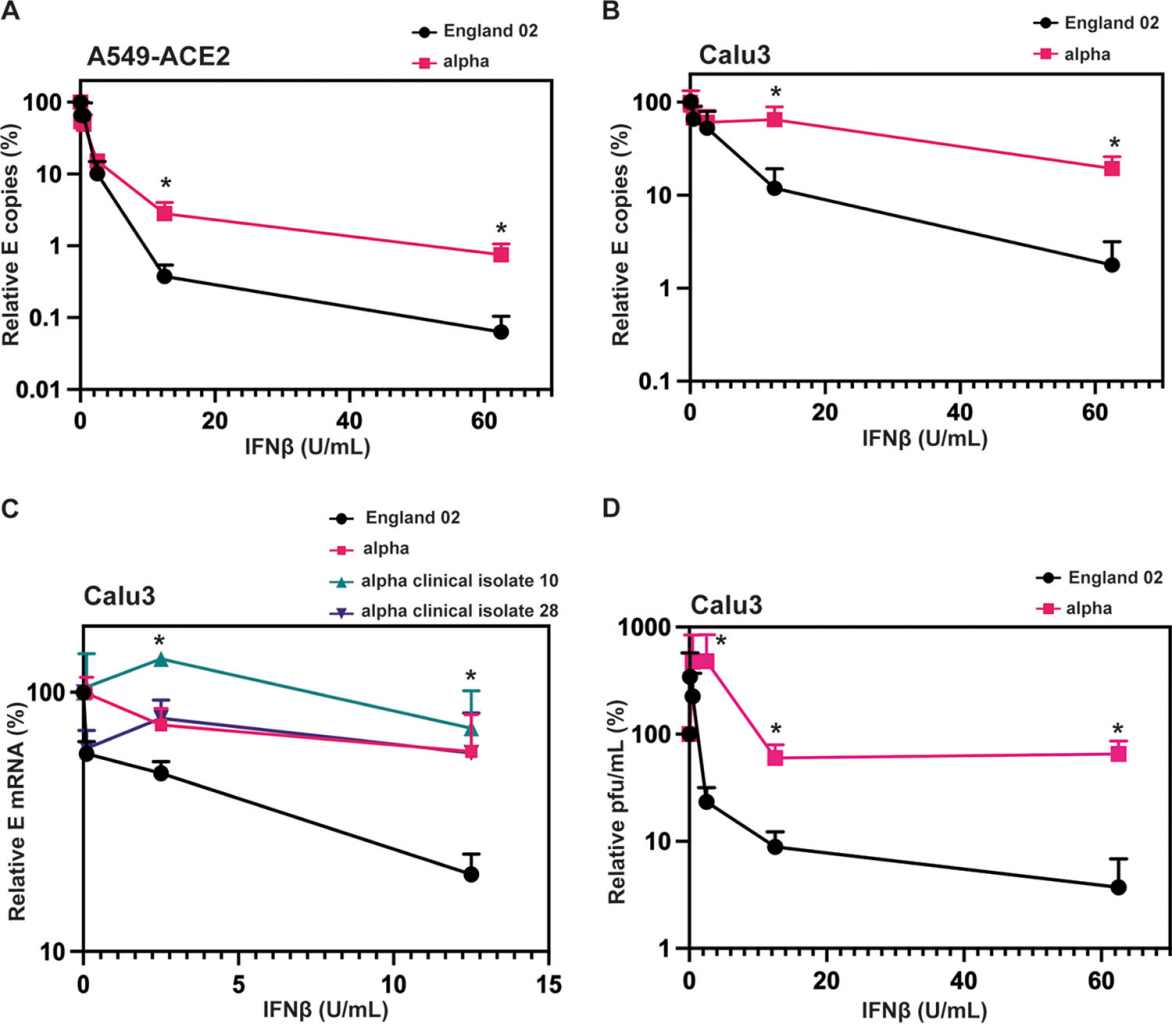
The alpha variant is resistant to IFN-β. (A) England-02 and alpha virus infection in A549-ACE2 cells pretreated with IFN-β. Cells were pretreated with increasing concentrations of IFN-β for 18 h prior to infection with either virus at 500 E mRNA copies/cell. Infection was quantified by RT-qPCR of E mRNA from the supernatant 48 h later and normalized to the untreated control. (B) England-02 and alpha virus infection in Calu-3 cells pretreated with IFN-β. Cells were pretreated with increasing concentrations of IFN-β for 18 h prior infection with either virus at 5,000 E copies/cell. Infection was quantified by RT-qPCR of E mRNA from the supernatant 48 h later and normalized to the untreated control. (C) England-02 and clinical isolates of alpha virus infection in Calu-3 cells pretreated with IFN-β and harvested as for panels A and B. Cells were pretreated with increasing concentrations of IFN-β for 18 h prior to infection with either virus at 5,000 E copies/cell. Infection was quantified by RT-qPCR of cellular E mRNA relative to GAPDH 48 h later and normalized to the untreated control. (D) Calu-3 cells were infected with England-02 or alpha as for panel B, and supernatant from infected cells was used to infect Vero-E6-TMPRSS2 cells for 72 h. The number of PFU per milliliter was determined by plaque assay. Data are means and SEM (*n* = 3). Statistics were calculated in Prism using a *t* test. *, *P* < 0.05 for the different viruses at individual IFN concentrations.

While previous data have indicated that the original Wuhan-like SARS-CoV-2 virus can delay pattern recognition of viral RNA in target cells, its replication is highly sensitive to exogenous IFN-I treatment in culture, in part determined by IFITM2 (30). Having confirmed that the alpha variant is resistant to IFITM expression when ectopically expressed, we then tested if alpha was also more resistant to the effects of IFN-β, as suggested by others (31, 32). Indeed, we found from measuring supernatant viral RNA 48 h after infection of A549-ACE2 cells that alpha is more resistant than England-02 to pretreatment with increasing doses of IFN-β (Fig. 5A). Additionally, this was recapitulated in lung epithelial Calu-3 cells, which naturally express ACE2 and TMPRSS2 (Fig. 5B). We further extended these observations to two clinical isolates of alpha (clinical isolates 10 and 28) (Fig. 5C) and measured viral RNA in cell lysates. This confirmed that two further clinical isolates of alpha grown from patient swabs are also resistant to pretreatment with IFN-β. Finally, we showed that the alpha isolate is resistant to exogenous IFN-β pretreatment by taking the supernatant from infected Calu-3 cells pretreated with IFN-β and measuring the viral infectivity by plaque assay on Vero-E6-TMPRSS2 cells, confirming that the alpha variant still actively replicates in the presence of IFN-β to produce infectious virions (Fig. 5D). Thus, in comparison to a representative example of Wuhan-1-like SARS-CoV-2, the alpha variant has a marked resistance to IFN-I.

### Discordance between the incorporation of furin-processed spike proteins into lentiviral particles and native virions.

It has been postulated that the P681R and P681H mutations that have emerged in the delta, alpha, and omicron variants enhance spike processing, which facilitates a more cell surface-based route of entry (33). However, whether the P681R or P681H mutations confer a greater degree of S processing has been debated (20, 34). We had previously identified cleavage at the S1/S2 boundary in the Wuhan-1 virus as a factor in reduced IFITM2 sensitivity and therefore postulated that the P681H mutation may lead to increased S1/S2 cleavage and explain why alpha is IFITM resistant in A549-ACE2s. PLV particles assemble and bud at the plasma membrane (35) and incorporate SARS-CoV-2 spike into virions, which reaches the cell surface by bulk anterograde transport because it escapes coatomer protein I (COPI)-mediated ER/Golgi retention (29), and this process is enhanced by removal of the C-terminal 19 amino acid (aa) of spike (36). In contrast, native CoV virions assemble at, and bud into, intracellular Golgi-derived membranes and are then secreted.

**FIG 6:**
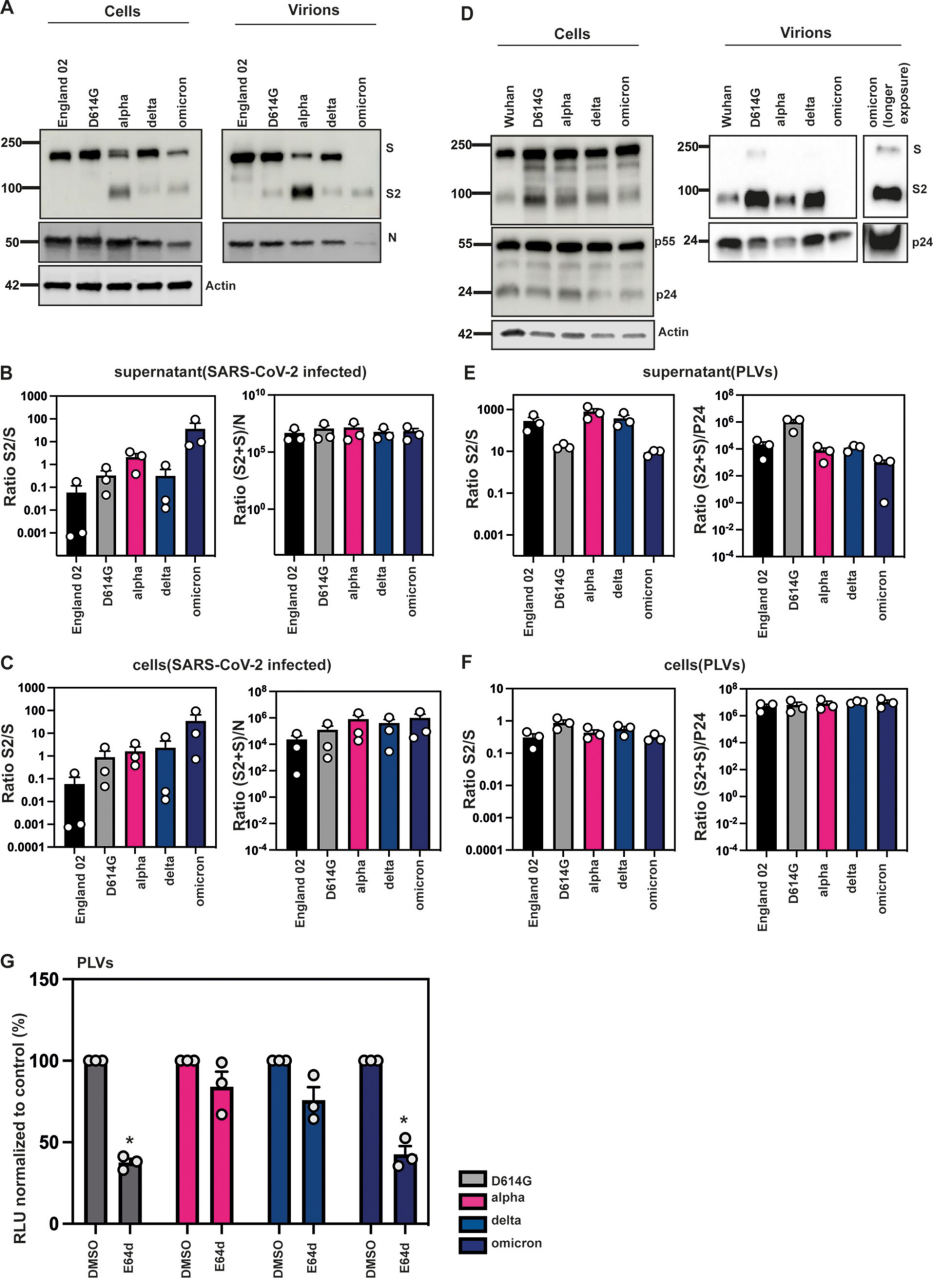
Spike is differentially cleaved across the major variants but not in PLVs. (A) Representative Western blot of spike protein in cell lysates and purified supernatants of infected Vero-E6-TMPRSS2 cells. Cells were infected with Wuhan, D614G, alpha, delta, or omicron isolates at an MOI of 1 for 30 h. Virions were purified through a 20% sucrose gradient. (B) Quantification of spike in cell lysates of infected Vero-E6-TMPRSS2 cells after 30 h. (C) Quantification of spike in purified supernatant from infected Vero-E6-TMPRSS2 cells after 30 h. (D) Representative Western blot of spike protein in cell lysates and purified supernatants from PLVs. PLVs were produced in 293T/17 cells and immunoblotted 48 h after transfection. Virions were purified through a 20% sucrose gradient. (E) Quantification of spike in cell lysates of 293T/17 cells used to produce PLVs. (F) Quantification of spike in purified PLVs produced in 293T/17 cells. (G) E64d sensitivity of D614G, alpha, delta, and omicron PLVs. A549-ACE2 cells were pretreated with 10 μM E64d for 1 h prior to transduction, and infection was quantified on the basis of luciferase activity 48 h later. Data are means and SEM (*n* = 3). Statistics were calculated in Prism using a *t* test. *, *P* < 0.05 between control and drug.

While most studies have compared the incorporation of furin-cleaved spike in PLVs to that of spike in lysates of SARS-CoV-2-infected cells, we compared S cleavage and incorporation into sucrose-pelleted virions for sequence-verified isolates of the major VOC and lentiviral pseudotypes made with the same spike (Fig. 6A to F). In contrast to the HEK293T cells producing PLVs, Vero-E6-TMPRSS2 cells infected with fixed doses of the Wuhan-1-like England-02, D614G, alpha, delta, and omicron isolates displayed marked differences in cleaved spike content in both cells and pelleted virions. Lysates of 30-h-infected Vero-E6-TMPRSS2 cells displayed markedly larger amounts of the S2 cleavage product as a proportion of uncleaved spike for the D614G mutant and the VOC than the England-02 isolate. While incorporation of spike into harvested virions (S levels in pelleted virions relative to N) was equivalent (Fig. 6A and C), virions produced from Vero-E6-TMPRSS2 reflected the cell lysate well, with alpha and omicron showing much higher relative cleaved spike incorporation than delta or D614G variants, which in turn was more pronounced than that of England-02 (Fig. 6A and B).

That these results contrast with data from other groups producing virus in other systems highlights the idea that the relative proportion of cleaved spike on SARS-CoV-2 virions is likely to be highly dependent on the cell line in which the virus is grown. In contrast, PLVs displayed clear differences with the native virus: while all spikes were similarly expressed in the cell lysates, there were clear differences in the level of PLV incorporation of between PLVs (6D-6F), indicating that PLVs may not give a true reflection of the spike incorporation or processed conformation on native virions. Discrepancies between lentiviral vectors and virus spike processing was also recently suggested by the Côté group (22), and it is likely that the cell type in which the viruses and PLVs are produced influences the observed spike processing and may explain some of the differences in the literature (22, 37). Given that the structural proteins E, M, and N are known to regulate S retention, assembly, and glycosylation (38), we suggest that differences in spike cleavage based solely on assays using spike-only PLVs be interpreted with caution. Furthermore, as demonstrated in Fig. 3, PLVs with a ΔCT result in both differential S1/S2 cleavage and cathepsin dependence, further confirming that this needs to be taken into account when determining consequences for spike cleavage.

Next, we tested if the alpha, delta, and omicron variants used the same route of entry given the polybasic cleavage site mutations. Other groups have suggested that the omicron variant, despite containing a P681H mutation, is more dependent on the endosomal route of entry because the receptor binding domain is more likely to be in the “down” conformation (27). This change in entry route may account for our observation that omicron retains IFITM2 sensitivity. We thus hypothesized that despite the P681H mutation, omicron, unlike alpha, would still require endosomal cathepsins for entry. To test this, we pretreated A549-ACE2 cells with the endosomal cathepsin inhibitor E64d and infected them with D614G, alpha, delta, and omicron PLVs. We found that, in line with what others have described, omicron displayed E64d sensitivity similar to that of the D614G mutant (Fig. 6G). The alpha or delta variants essentially showed no significant dependence on cathepsin-mediated S cleavage relative to the D614G variant. Overall, these results suggest that S1/S2 cleavage is highly cell-type dependent and does not necessarily correlate with viral entry route.

### The P681H mutation is necessary for conferring IFITM and IFN-β resistance in alpha by promoting a cell surface route of viral entry.

**FIG 7:**
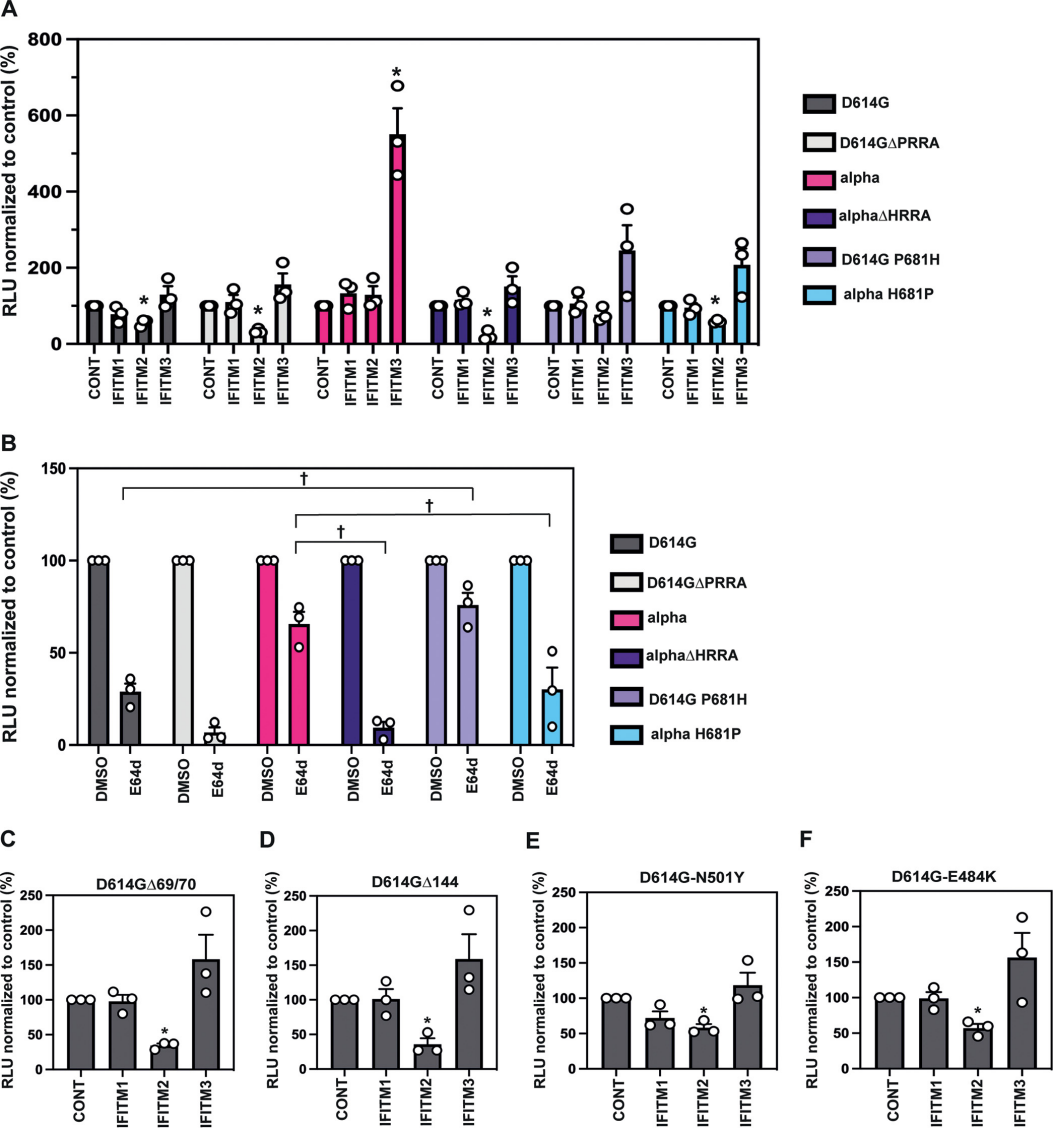
The P681H mutation confers IFITM resistance to a wild-type spike and a reduced dependence on E64d. (A) D614G, D614GΔPRRA, alpha, alpha-ΔHRRA, D614G P681H, and alpha H681P and PLV infection in A549-ACE2 cells stably expressing the individual IFITMs. PLV entry was quantified on the basis of luciferase activity 48 h later and normalized to control values. (B) E64d treatment of A549-ACE2 cells infected with PLVs. A549-ACE2s were pretreated with 10 μM E64d prior to transduction with D614G, alpha, delta, or omicron PLVs, and infection was quantified on the basis of luciferase activity 48 h later. (C to F) PLVs of individual mutations from alpha in the D614G background were used to infect A549-ACE2-IFITM cells, and infection was quantified on the basis of luciferase activity 48 h later. Infection normalized to control values from cells with no IFITM is shown. Data are means and SEM (*n* = 3). Statistics were calculated in Prism using ANOVA. Asterisks and daggers indicate significance (*P* < 0.05) between control cells and individual IFITMs or drug and between different IFITM/drug conditions.

Our previous data indicated that IFITM sensitivity of SARS-CoV-2 spike can be increased by deletion of the polybasic cleavage site (14). Given that the alpha spike acquired the P681H mutation and we demonstrated that it is relatively insensitive to an inhibitor of endosomal entry (Fig. 6G), we hypothesized that P681H might be a determinant of resistance to IFN and IFITMs for the alpha spike. Using PLVs on A549-ACE2-IFITM cells, we first confirmed that ablation of the entire polybasic cleavage site increases IFITM2 sensitivity of the D614G mutant, as we have previously demonstrated for the Wuhan-1 spike (14). As expected, D614GΔPRRA is highly sensitive to IFITM2 and is not cleaved on PLV particles (Fig. 7A; Fig. S3C to E). Next, we tested if the same polybasic cleavage site deletion sensitized alpha to the IFITMs. Not only was the ΔHRRA mutant sensitive to IFITM2, but also, we abolished the IFITM3 enhancement phenotype observed with the alpha PLV (Fig. 7A; additional statistics are provided in Fig. S3A and B), suggesting that prior S1/S2 cleavage was essential for both of these phenotypes.

Having confirmed that alpha spike could be sensitized to IFITM2 by deletion of the HRRA site, we next tested whether the P681H mutation alone could confer IFITM resistance to D614G S, and vice versa. We found that the P681H mutation in the D614G background was sufficient to abolish IFITM2 sensitivity but was not able to confer the level of IFITM3-mediated enhancement that we observe with alpha. However, the H681P mutation in alpha sensitized the alpha PLV to IFITM2, although not to the same extent as the ΔHRRA mutation, and also reduced the IFITM3 enhancement of alpha. We noted that the H681P mutation did not revert the cleavage of the alpha spike in the context of PLVs (Fig. S3C to E); however, as suggested in Fig. 5, conclusions regarding spike cleavage drawn from observations with PLVs may not represent the real virus. We concluded that although the P681H mutation is necessary for IFITM resistance, it is likely that other contextual mutations in the alpha spike are required for this to be sufficient for IFITM3 enhancement. Next, we tested whether the P681R mutation in the D614G background alters IFITM sensitivity (Fig. S3F). Unlike the P681H mutation, the P681R mutation did not alter the IFITM sensitivity of the D614G virus. Additionally, reverting R861 to a P in the delta S had little impact on IFITM sensitivity (Fig. S3G), further suggesting that the P681R mutation itself cannot confer IFITM resistance.

We then wanted to confirm whether the P681H mutation confers IFITM resistance by reducing the dependence on endosomal entry in the A549-ACE2 system. Previously, we demonstrated that the alpha spike is relatively insensitive to the effects of the cathepsin inhibitor E64d. To test whether the increased IFITM sensitivity of the ΔHRRA and H681P mutants correlated with increased endosomal entry and therefore exposure to IFITM2, we pretreated cells with the cathepsin inhibitor E64d as before and infected them with PLVs (Fig. 7D). As expected, we found that the proteins with D614G and alpha polybasic cleavage site deletions were highly sensitive to E64d. Additionally, the H681P mutation conferred increased E64d sensitivity to alpha, suggesting that this mutant is more reliant on cathepsin-dependent entry and therefore more likely to encounter IFITM2 (Fig. 7B). As expected, the inverse P681H mutation in the context of D614G conferred decreased E64d sensitivity to the wild-type spike. This suggests that the P681H mutation alone is sufficient to confer increased preference for cell surface entry to a D614G-bearing PLV. In the context of the alpha spike, we further suggest that the P681H mutation is a determinant of route of viral entry and therefore of sensitivity to antiviral proteins that occupy endosomal compartments. Having established that the P681R mutation did not alter IFITM sensitivity, we hypothesized that this mutation alone would not reduce E64d sensitivity to a wild-type spike. Indeed, while the P681H mutation reduces cathepsin-dependence, the P681R mutation is indistinguishable from D614G in terms of E64d sensitivity (Fig. S3H). This suggests that the P681R mutation does not confer cell surface-mediated entry in the A549-ACE2 cells. Finally, to determine whether any of the other defining mutations in the alpha spike altered IFITM sensitivity, we generated single mutants with the Δ69/70 (Fig. 7C), Δ144 (Fig. 7D), N501Y (Fig. 7E), and E484K (Fig. 7F) mutations, which emerged in several sublineages. None of these mutations significantly altered IFITM resistance.

### Reversion of the P681H mutation sensitizes the alpha variant to IFN-β and IFITM2.

**FIG 8:**
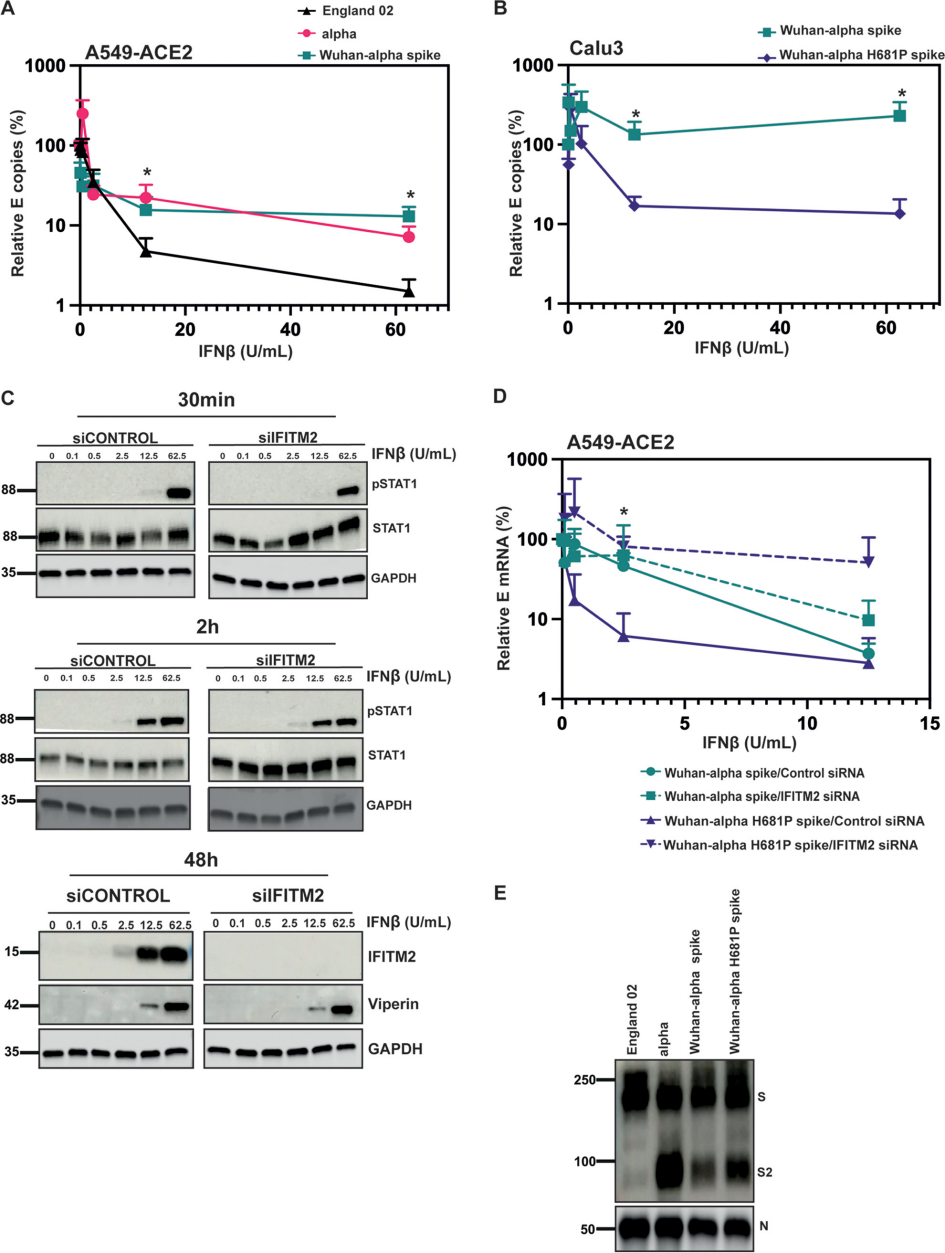
The P681H mutation is necessary and sufficient for IFN-β resistance. (A) England-02, alpha, and Wuhan(alpha spike) virus infection in A549-ACE2 cells pretreated with IFN-β. Cells were pretreated with increasing concentrations of IFN-β for 18 h prior to infection with either virus at 500 E copies/cell. Infection was quantified by RT-qPCR of E mRNA in the supernatant 48 h later and normalized to the value for the mock-treated control. (B) Wuhan(alpha spike) and Wuhan(alpha spike H681P) virus infection in Calu-3 cells pretreated with IFN-β. Cells were pretreated with increasing concentrations of IFN-β for 18 h prior to infection with either virus at 5,000 E copies/cell. Infection was quantified by RT-qPCR of E mRNA in the supernatant 48 h later and normalized to the value for the mock-treated control. (C) Representative immunoblot of pSTAT1 and STAT1 in cell lysates with IFITM2 knocked down or a nontargeting control and subsequently treated with IFN-β. A549-ACE2 cells were transfected with siRNAs against nontargeting control or IFITM2 and then either treated with IFN-β for 30 min or 2 h and immunoblotted for pSTAT1 and STAT1 or treated for 24 h and blotted for viperin and IFITM2. (D) A549-ACE2 cells were transfected with siRNAs against nontargeting control or IFITM2 for 24 h and then treated with IFN-β for 18 h prior to infection with Wuhan(alpha spike) or Wuhan(alpha spike H681P) at 500 copies/cell. Infection was quantified by RT-qPCR of E gene relative to GAPDH 48 h later. Data are means and SEM (*n* = 3). Statistics were calculated in Prism using a *t* test. *, *P* < 0.05 for differences between the different viruses. (E) Representative immunoblot of England-02, alpha, Wuhan(alpha spike), and Wuhan(alpha spike) H681P viral stocks. England-02, alpha, Wuhan(alpha spike), and Wuhan(alpha spike H681P) viruses were purified through 20% sucrose and immunoblotted for spike and N proteins.

Finally, we tested if the H681P reversion was sufficient to revert the overall IFN-β resistance phenotype of alpha. We constructed a recombinant molecular clone of SARS-CoV-2 Wuhan-1 encoding spike from the alpha variant. This virus essentially mimicked the resistance of the alpha variant itself to IFN-β in comparison to England-02, demonstrating that the alpha spike alone is sufficient to confer a level of IFN-I resistance in A549-ACE2 cells (Fig. 8A). Then, we reverted the amino acid residue H681 in this recombinant virus to a proline. Importantly, this single point mutation was sufficient to confer a significant sensitivity to IFN-β in Calu-3 cells, indicating that it was a major determinant of IFN resistance in alpha spike (Fig. 8B). Furthermore, we wanted to confirm whether small interfering RNA (siRNA) knockdown of IFITM2 was sufficient to rescue the Wuhan(B.1.1.7 spike H681P) virus from IFN-β-mediated inhibition. We confirmed that IFITM2 knockdown had no effect on expression of other interferon-stimulated genes (ISGs) and IFN-β signaling, measured by STAT1 phosphorylation and viperin expression (Fig. 8C). We showed that the H681P-reverted virus was rescued from IFN-β restriction by IFITM2 knockdown, while the Wuhan(B.1.1.7 spike) virus was unaffected, consistent with this virus being resistant to IFITM restriction (Fig. 8D). Thus, this confirmed that the S protein of the alpha variant of SARS-CoV-2 is a determinant of type-I IFN resistance, which is primarily modulated by IFITM2. Most importantly, the P681H mutation is necessary for this. Interestingly, when we immunoblotted purified virions of the Wuhan(B.1.1.7 spike) and Wuhan(B.1.1.7 spike-H681P), we found that, similar to the PLVs (Fig. 6B and C), the H681P reversion did not affect the cleavage of the alpha S (Fig. 8E). Thus, P681H adaptive mutation is a determinant of IFN type I resistance acquired in the alpha variant that evades IFITM2 restriction. It further appears to exert its activity independently of S1/S2 cleavage by altering the route of viral cell entry.

## DISCUSSION

Here, we show that the spike protein of the alpha variant of SARS-CoV-2 is a determinant of viral resistance to IFN-I. This maps to the histidine residue adjacent to the polybasic cleavage site that has been mutated from the parental proline. While this has been shown to enhance spike cleavage at the S1/S2 boundary in a context-dependent manner (39), the H at this position in alpha rather than the cleavage itself appears to confer the IFN resistance phenotype. This is reinforced by the finding that deleting the last 19 amino acids of D614G spike results in enhanced S1/S2 cleavage but not enhanced IFITM resistance, further suggesting that cleavage *per se* is not the determining factor of the alpha variant’s IFITM resistance. The P681H mutation correlates with the abolition of the residual sensitivity to endosomal cathepsin inhibitors, implying a change in viral entry route that distinguishes alpha from delta. This residue is also necessary to confer both resistance to IFITM2 and enhancement by IFITM3 and, as we demonstrated in our previous study (14), confirms that the polybasic cleavage site can modulate IFITM entry restriction. Furthermore, we demonstrate that this mutation alone in a wild-type D614G spike is sufficient to promote reduced IFITM sensitivity, while the delta P681R mutation is not. Furthermore, we note that infection by alpha is enhanced in the presence of IFITM3, and this is abolished by cyclosporine H, cytoplasmic tail deletion, and the H681P mutation. IFITM3 was reported previously to enhance the entry of the coronavirus OC43 and more recently was suggested to enhance the entry of hepatitis B and D viruses (24, 40). Although it is surprising that an antiviral protein can enhance infection, this phenotype in multiple viruses suggests a common mechanism of hijacking host factors for viral entry. We also saw enhancement by IFITM1 or IFITM2 a factor of 1 or 2 with the alpha virus and PLVs to a variable degree. We suggest that this may be a factor of the trafficking of IFITMs through multiple compartments and the occasional presence of IFITM1 and -2 in the compartment where IFITM3 usually resides. Enhancement of coronavirus entry by the mutant IFITM1Δ117-125 has been documented, suggesting that IFITM1 can enhance viral entry depending on localization (41). We also showed previously that the Y19F mutation in IFITM2 results in enhancement of Wuhan entry, further suggesting that IFITM localization can alter its capacity to enhance coronavirus infection (14).

We suggest that the P681H change in alpha changes the site of viral fusion, thus avoiding the endosomal compartment where IFITM2 predominantly resides. Consistent with this, we showed that the alpha spike in a PLV is less sensitive to the cathepsin inhibitor E64d. Thus, we propose that these changes in the alpha spike have, in part, arisen to resist innate immunity. At least two studies suggest that variants of SARS-CoV-2 have begun to evolve further resistance to interferon-induced innate immunity (31, 42, 43). In one, viral isolates obtained over the course of the pandemic showed a reduced sensitivity to type I interferons in culture (42); in a second, the alpha variant showed a significantly reduced propensity to trigger pattern recognition in epithelial cells by cytoplasmic RNA sensors (31, 43). In contrast, another study found no difference in IFN sensitivity of the new variants in African green monkey Vero-E6 cells (11), although species specificity in viral sensitivity to ISGs is a well-characterized trait that could explain this discrepancy (44). The SARS-CoV-2 genome contains multiple mechanisms to counteract host innate immune responses, and much remains to be learned about the mechanisms deployed by this virus and its relatives. While many reports on SARS-CoV-2 evolution have naturally focused on the pressing concern of the potential for vaccine escape, it is very unlikely that all selective adaptations that we see arising in VOC can be due solely to escape from adaptive immunity. The alpha spike, for example, displays only a minor reduction in sensitivity to neutralizing antibodies (NAbs) (8, 45–47). However, this VOC had a considerable transmission advantage, giving rise to the suspicion that it may have arisen in an immunocompromised individual with a persistent infection, providing ample time for changes to be selected that further evade innate immunity, including those that target viral entry (48, 49).

In terms of IFITM resistance of VOC spike proteins, so far, we have seen a marked change in phenotype only in the alpha variant. This is despite the fact that both delta and omicron, variants that superseded alpha, also showed an adaptation for enhanced S1/S2 cleavage with a P681R and P681H change, respectively (20, 39). This suggests that cleavage of S1/S2 is necessary but not sufficient for IFITM resistance and that other mutations in each cognate spike act in concert to determine relative IFITM sensitivity. Despite the increased cleavage of a D614G-containing isolate in delta and omicron relative to England-02, these viruses are not IFITM resistant. This suggests that the P681H mutation confers IFITM resistance through a mechanism distinct from S1/S2 cleavage itself. We and others show that omicron is sensitive to E64d inhibition, and we suggest that this preference for endosomal entry correlates with omicron’s IFITM sensitivity (27).

The spike of omicron contains 30 mutations, 12 of which are in the RBD and have been suggested to increase the affinity for ACE2 (50). The constellation of mutations in the RBD of omicron also promotes an “RBD down” closed conformation, which necessitates cathepsin-mediated cleavage in the endosome rather than surface TMPRSS2-mediated cleavage (27). This suggests that the up conformation of the RBD is required for H681 to exert its IFN resistance phenotype (27). Furthermore, omicron contains an H655Y mutation, which has been suggested to enhance endosomal entry (51). Despite delta containing a P681R mutation, we report that this spike is not IFITM resistant, nor is the kappa variant, which also bears a P681R mutation and was relatively short-lived as a variant. Despite the delta spike demonstrating E64d insensitivity, the P681R mutation alone does not result in reduced IFITM sensitivity or decreased E64d sensitivity to a D614G spike. This implies that there are other factors besides the P681R mutation governing delta’s route of viral entry. Two recent papers have suggested that certain matrix metalloproteinases (MMPs) can mediate an alternative route of entry to TMPRSS2, and that this can be utilized by the delta variant (22, 52). It is possible that a combination of these viral entry routes is variably present in different cell types and may therefore explain differential IFITM sensitivities of VOC.

Finally, the delta variant also contains different RBD mutations than alpha, in particular the T478K and L452R mutations, which may also affect the RBD conformation and be a factor in delta’s relative sensitivity to IFITMs. The mutations in the RBDs of delta and omicron have led to hypotheses that both of these variants were driven by antibody escape, suggesting that selection pressures on the alpha variant may have been more due to innate immunity. It is important to note that the discordance between virion-incorporated spike species in the native SARS-CoV-2 particle and lentiviral pseudotypes imply a degree of cell type dependency as well as cellular location of viral assembly in the relative presence of cleaved spike. We also demonstrate that this issue is of particular concern for those using C-terminal deletions of the COPI retention signal in spike. We would hesitate to ascribe some of the phenotypes associated with VOC spike protein simply to differences in furin cleavage efficiency or phenotypes observed with Δ19aa PLVs when the route of viral entry is implicit to the phenotype.

Viral glycoproteins are dynamic structures that shift through large-scale conformational changes while interacting with their cognate receptors mediating viral membrane fusion (53). Such context dependency is therefore likely to be complex and will arise under competing selective pressures. Indeed, we showed previously that the HIV-1 envelope glycoprotein of transmitted viruses is IFITM insensitive and that this contributes to their overall type I IFN resistance (54). As HIV-1 infection progresses over the first 6 months in an infected person, the circulating variants increase in IFN/IFITM sensitivity, and this is determined by adaptive changes in Env that resist the early neutralizing antibody response (55). Such escape has structural and functional implications for such dynamic proteins that may impact receptor interactions and route of entry into the target cell.

The mapping of IFN-I resistance to P681H to the polybasic cleavage site of alpha, combined with the observation of reversion of IFN-I sensitivity by the restoration of the P without affecting the cleavage of virion-associated spike, suggests that H681 exerts its effects on viral entry and IFITM/IFN-I sensitivity downstream of cleavage itself. While it is possible that this could be simply related to stability of the cleaved form, it is intriguing that the C-terminal RRAR of S1 has also been proposed as a ligand for neuropilin-1 (NRP-1), a receptor for furin-processed growth factors like vascular endothelial growth receptor A (VEGF-A). NRP-1 was found to promote the entry and replication of SARS-CoV-2 in an FCS-dependent manner (56, 57). Given the accumulating evidence that interprotomer interactions in the S trimer affect the accessibility of cleavage sites in spike (58), future studies will determine whether a role for NRP-1 in entry also governs sensitivity to IFITM restriction and IFN sensitivity.

While the polybasic cleavage site of the SARS-CoV-2 S reduces its IFITM sensitivity, other interferon-induced proteins may contribute to this phenotype. The guanylate binding protein family, and particularly GBP2 and GBP5, has been shown to have a general antiviral activity against enveloped viruses by dysregulating furin processing of diverse viral and cellular proteins (59). Similarly, IFITM overexpression in HIV-infected cells can lead to their incorporation into virions and in some cases promote defects in glycoprotein incorporation (60). Future studies will confirm whether either of these mechanisms is involved in the IFN resistance associated with the P681H mutation in alpha (27).

In summary, the spike protein of SARS-CoV-2 alpha increases resistance to IFN-I, and this correlates with the P681H mutation. Furthermore, it also correlates with IFITM resistance, as IFITM2 knockdown rescues the IFN-sensitive alpha H681P virus, but not alpha. Despite also containing P681R/P681H mutations, the delta and omicron variants are not IFITM resistant in the A549-ACE2 system. We suggest that factors such as spike conformation and alternate routes of viral entry all act in concert to determine the relative sensitivities of spike proteins to antiviral proteins that affect viral entry.

## MATERIALS AND METHODS

### Cells and plasmids.

HEK293T-17 (ATCC; CRL-11268), Calu-3 (ATCC; HTB-55), A549-ACE2, Vero-E6, Vero-E6-TMPRSS2, and A549-ACE2 cells expressing the individual IFITM proteins were cultured in Dulbecco’s modified Eagle medium (DMEM; Gibco) with 10% fetal bovine serum (FBS; Invitrogen) and 200 μg/mL gentamicin (Sigma), and incubated at 37°C and 5% CO_2_. Cells stably overexpressing ACE2, TMPRSS2, and IFITM were generated as previously described (14).

Codon-optimized SARS-CoV-2 Wuhan spike and ACE2 were kindly provided by Nigel Temperton. Codon-optimized variant spikes (alpha and beta) were kindly provided by Katie Doores. Codon optimized variant spikes (gamma, kappa, delta) were kindly provided by Wendy Barclay. Plasmid containing the TMPRSS2 gene was kindly provided by Caroline Goujon. Spike mutants were generated with Q5 site-directed mutagenesis kit (E0554) following the manufacturer’s instructions and using the following forward and reverse primers: D614G (GCTGTACCAGGGCGTGAATTGCA, ACGGCCACCTGATTGCTG), B.1.351. Δ242-244 (ATTTCATATCTTACACCAGGC, ATGCAGGGTCTGGAATCTG), D614G P681H (GACCAATAGCCACAGAAGAGCCAGAAGC, TGGGTCTGGTAGCTGGCG), B117 ΔHRRA (AGAAGCGTGGCCAGCCAG, GCTATTGGTCTGGGTCTGGTAG), B117 H681P (GACCAATAGCcccAGAAGAGCCAG, TGGGTCTGGTAGCTGGCG), Δ69/70 (AGCGGCACCAATGGCACC, GATGGCGTGGAACCAGGTC), Y144 (CATAAGAACAACAAGAGC, ATAAACACCCAGGAAAGG), E484K (TAATGGCGTGAAGGGCTTCAATTGCTACTT, CACGGTGTGCTGCCGGCC), N501Y (CCAGCCTACCTACGGCGTGGGCT, AAGCCGTAGCTCTGCAGAG), and ΔCT (GTCCTGCTGCTGATGAGACGAGGACGACAGCG, CCACACGAACAACACCCT).

Stable A549 cell lines expressing ACE2 (pMIGR1-puro) and IFITMs (pLHCX) were generated and selected as described previously (14).

### Production of PLVs and infection.

HEK293T-17 cells were transfected with firefly luciferase-expressing vector (CSXW), HIV gag-pol (8.91), and spike plasmid with PEI-max as previously described (14). One hundred microliters of viral supernatant was then used to transduce each cell line of interest, and readout was measured as luciferase activity 48 h later (Promega Steady-Glo [E2550]).

### Cyclosporin H assay.

Cells were pretreated with 30 μM cyclosporine H (Sigma; SML1575) for 18 h. Cells were then infected with PLVs as described above, and viral entry was quantified on the basis of luciferase activity 48 h later.

### E64d assay.

A549-ACE2 cells were pretreated with 10 μM E64d (Sigma; E8640) for 1 h at 37°C prior to infection. Cells were transduced with PLVs, and infection was determined on the basis of luciferase activity 48 h later.

### Passage and titration of SARS-CoV-2.

PHE England strain 02/2020 and D614G isolate were propagated in Vero-E6-TMPRSS2 cells, and titer was determined by plaque assay (14). Plaque assays were performed by infecting Vero-E6-TMPRSS2 cells with serial dilutions of SARS-CoV-2 for 1 h. Subsequently, 2× overlay medium (DMEM with 2% FBS and 0.1% agarose) was added, and infected cells were fixed with 4% paraformaldehyde (PFA) 72 h after infection and stained with crystal violet. Plaques were counted and multiplicity of infection calculated for subsequent experiments. A replication-competent alpha variant was kindly provided by Wendy Barclay (Imperial College London) (61). The spike gene sequences of all virus stocks were confirmed at each passage to ensure no loss of the polybasic cleavage site.

### Generation of recombinant full-length viruses.

We used the previously described method of transformation-associated recombination (TAR) in yeast (62), with some modifications, to generate the mutant viruses described in this study. Briefly, a set of overlapping cDNA fragments representing the entire genomes of SARS-CoV-2 Wuhan isolate (GenBank accession no. MN908947.3) and the B.1.1.7 alpha variant were chemically synthesized and cloned into pUC57-Kan (Bio Basic Canada Inc. and Genewiz, respectively). The cDNA fragment representing the 5′ terminus of the viral genome contained the bacteriophage T7 RNA polymerase promoter preceded by a short sequence stretch homologous to the XhoI-cut end of the TAR-in-yeast vector pEB2 (63). The fragment representing the 3′ terminus contained the T7 RNA polymerase termination sequences followed by a short segment homologous to the BamHI-cut end of pEB2.

To generate Wuhan virus carrying the alpha variant spike, a mixture of the relevant synthetic cDNA fragments of the Wuhan and alpha variants was cotransformed with XhoI-BamHI-cut pEB2 into the Saccharomyces cerevisiae strain TYC1 (*MAT*α *ura3-52 leu2*Δ*1 cyh2*^r^, containing a knockout of DNA Ligase 4) (63) that had been made competent for DNA uptake using the LiCl_2_-based yeast transformation kit (YEAST1-1KT; Merck). The transformed cells were plated on minimal synthetic defined (SD) agar medium lacking uracil (Ura) but containing 0.002% (wt/vol) cycloheximide to prevent selection of cells carrying the empty vector. Following incubation at 30°C for 4 to 5 days, colonies of the yeast transformants were screened by PCR using specific primers to identify those carrying plasmids with fully assembled genomes. Selected positive colonies were then expanded to grow in 200 mL SD-Ura dropout medium, and the plasmid was extracted. Approximately 4 μg of the extracted material was then used as the template to synthesize viral genomic RNA transcripts *in vitro* using the Ribomax T7 RNA transcription kit (Promega) and Ribo m7G cap analogue (Promega) as per the manufacturer’s protocol. Approximately 2.5 μg of the *in vitro*-synthesized RNA was used to transfect ~6 ×10^5^ BHK-hACE2-N cells stably expressing the SARS-CoV-2 N and the human ACE2 genes (64) using the MessengerMax lipofection kit (Thermo Scientific) as per the manufacturer’s instructions. Cells were then incubated until signs of viral replication (syncytium formation) became visible (usually after 2 to 3 days), at which time the medium was collected (P0 stock) and used further as a source of rescued virus to infect Vero-E6 cells to generate P1 and P2 stocks. Full genome sequences of viruses collected from P0 and P1 stocks were obtained in order to confirm the presence of the desired mutations and exclude the presence of other spurious mutations. Viruses were sequenced using Oxford Nanopore Technologies as previously described (65).

To generate Wuhan virus carrying the alpha spike gene with the H681P mutation, we first introduced this mutation into the relevant alpha variant cDNA fragment by site-directed mutagenesis. This fragment was combined with those described above, and the mixture was then used to generate plasmid pEB2 carrying the cDNA genome of Wuhan encoding the H681P alpha spike by the TAR-in-yeast procedure. The virus rescue and subsequent characterization were performed as described above.

### Isolation and propagation of clinical viral isolates.

Viruses were isolated on Vero-E6 cells (ATCC; CRL 1586) from combined naso-oropharyngeal swabs submitted for routine diagnostic testing by real-time reverse transcription-PCR (RT-PCR) and shown to be from the B.1.1.7 (alpha) variant by on-site whole-genome sequencing (Oxford Nanopore Technologies, Oxford, UK) (66). Infected cells were cultured at 37°C and 5% CO_2_ in DMEM (Gibco, Thermo Fisher, UK) supplemented with 2% FBS (Merck, Germany), penicillin-streptomycin, and amphotericin B.

All work performed with full-length SARS-CoV-2 preparations, as well as isolation and propagation of viral isolates from swabs, was conducted inside a class II microbiological safety cabinet in a biosafety level 3 (BSL3) facility at King’s College London.

### Infection with replication competent SARS-CoV-2.

A549-ACE2 cells (1.5 × 10^5^) were infected for 1 h at 37°C with SARS-CoV-2 replication-competent viruses at a multiplicity of infection (MOI) of 0.01 or 500 E gene mRNA copies/cell. Calu-3 cells (2 × 10^5^) were infected for 1 h at 37°C with SARS-CoV-2 replication-competent viruses at 5,000 E gene mRNA copies/cell. Medium was replaced, and cells were incubated for 48 h at 37°C, after which cells or supernatant was harvested for RNA extraction or protein analysis.

### Intracellular N staining.

A549-ACE2 IFITM cells (1.5 × 10^5^) were infected for 1 h at 37°C with SARS-CoV-2 replication-competent VOC to achieve the same percentage of infected cells as under the mock-infection condition. After 24 h infection, cells were trypsinized and fixed with 4% PFA during 30 min at room temperature. Cells were permeabilized with 1× phosphate-buffered saline (PBS) plus 0.5% Triton during 10 min following blocking with 5% FBS in 1× PBS for 20 min. After blocking, cells were stained with anti-N antibody (CR3009, mouse) for 45 min at room temperature and washed once with 1× PBS. Next, cells were incubated with secondary anti-mouse immunoglobulin conjugated to Alexa Fluor 488 for 25 min. Finally, cells were washed with 1× PBS and analyzed on a BD FACSCanto II flow cytometer using FlowJo software.

### Interferon assays.

Cells were treated with different doses of IFN-β (PBL Assay Science; 11415-1) for 18 h prior to infection. The following day, medium was replaced, and the infection was performed as described above. Viral RNA levels in cells or supernatants were measured 48 h after infection by RT-qPCR.

### siRNA knockdown of IFITM2.

A549-ACE2 cells were reverse transfected using 20 pmol of nontargeting siRNA (D-001206-13-20) or IFITM2 siRNA (M-020103-02-0010) with 1 μL of RNAiMax (Invitrogen). Cells were incubated for 24 h prior to a second round of reverse transfection. Eight hours later, cells were treated with different doses of IFN-β. Following 18 h of IFN treatment, cells were infected with full-length viruses as previously described.

### RT-qPCR.

RNA from infected cells was extracted using a Qiagen RNeasy minikit (Qiagen; 74106) following the manufacturer’s instructions. One microliter of each extracted RNA was used to performed one-step RT-qPCR using TaqMan Fast Virus one-step master mix (Invitrogen). The relative quantities of envelope (E) gene were measured using a SARS-CoV-2 (2019-nCoV) CDC qPCR probe assay (Integrated DNA Technologies [IDT]). Relative quantities of E gene were normalized to GAPDH mRNA levels (Applied Bioscience; Hs99999905_m1).

Supernatant RNA was extracted using RNAdvance viral XP (Beckman) following the manufacturer’s instructions. Five microliters of each RNA was used for one-step RT-qPCR (TaqMan Fast Virus one-step master mix) to measured relative quantities of E and calibrated to a standard curve of E kindly provided by Wendy Barclay.

### SDS-PAGE and Western blotting.

Cellular samples were lysed in reducing Laemmli buffer at 95°C for 10 min. Supernatant or viral stock samples were centrifuged at a relative centrifugal force (RCF) of 18,000 through a 20% sucrose cushion for 1 h at 4°C prior to lysis in reducing Laemmli buffer. Samples were separated on 8 to 16% Mini-Protean TGX precast gels (Bio-Rad) and transferred onto nitrocellulose membranes. Membranes were blocked in milk or Bovine serum albumin (BSA) prior to detection with specific antibodies: 1:1,000 ACE2 rabbit (Abcam; Ab108209), 1:5,000 GAPDH rabbit (Abcam; Ab9485), 1:2,000 anti-GAPDH mouse (Proteintech; 60004-1-Ig), 1:5,000 HSP90 mouse (GeneTex; Gtx109753), 1:50 HIV-1 p24Gag mouse (67), 1:1,000 spike mouse (GeneTex; Gtx632604), 1:1,000 anti-SARS-CoV-2 N rabbit (GeneTex; GTX135357), 1:1,00 anti-pSTAT1 mouse (BD Transduction Laboratories; 612133), 1:1,000 anti-STAT1 rabbit (Cell Signaling; 9172S), and 1:1,000 anti-viperin mouse (Millipore; MABF106). Proteins were detected using LI-COR and ImageQuant LAS 4000 cameras.

### Ethics.

Clinical samples were retrieved by the direct care team in the Directorate of Infection, at St Thomas Hospital, London, United Kingdom, and anonymized before being sent to the King’s College London laboratories for virus isolation and propagation. Sample collection and studies were performed in accordance with the UK Policy Framework for Health and Social Care Research and with specific Research Ethics Committee approval (REC 20/SC/0310).
