# Supplementary material for: The P681H Mutation in the Spike Glycoprotein of the Alpha Variant of SARS-CoV-2 Escapes IFITM Restriction and Is Necessary for Type I Interferon Resistance
Source: J Virol. 2022 Nov 9;96(23):e01250-22. doi: 10.1128/jvi.01250-22 (PMC9749455; doi:10.1128/jvi.01250-22)
Supplement: Supplemental file 1 — Fig. S1 to S3. Download jvi.01250-22-s0001.pdf, PDF file, 3.4 MB [file jvi.01250-22-s0001.pdf]

Supplementary Figure 1. Cyclosporin H treatment abolishes IFITM3 enhancement. A) Relative titre of VOC PLVs on A549-ACE2 cells. A549-ACE2 were transduced with PLVs of Wuhan, D614G, alpha, beta, gamma, kappa, delta and omicron for 48h and infectivity quantified by Luciferase activity. B) Representative immunoblot of A549-ACE2 cells stably expressing IFITMs 1, 2 and 3. **C, D)** D614G PLVs pre-treated with Cyclosporin H. A549-ACE2s stably expressing the individual IFITMs were pre-treated with 30 µM of Cyclosporin H for 18 hours prior to infection with D614G PLVs. Infection was quantified by Luciferase activity 48 hours after infection and normalized to control cells. Data shown are mean ± SEM, n=3. Statistics were calculated in Prism using *t*-test, stars indicate significance between IFITM3 mock and IFITM3 CsH (*P=<0.05). **E)** Western blots of IFITM-expressing A549 cells treated for 18h with 30 µM of Cyclosporin H.

Supplementary Figure 2. The delta and omicron viruses are IFITM sensitive in A549-ACE2s. A) Infection of A549-ACE2 stably expressing the individual IFITMs with delta virus at MOI 0.01. Infection was quantified by RT-qPCR of E gene relative to GAPDH 48 hours later; graph represents E mRNA levels relative to GAPDH. B) intracellular N staining by flow cytometry of A549-ACE2 IFITM cells infected with omicron virus. A549-ACE2 expressing the individual IFITMs were infected with an omicron isolate for 48h. Infection was measured by percentage of N positive cells by flow cytometry. Data analysed in FlowJo**.** Data shown are mean ± SEM, n=3. Statistics were calculated in Prism using *t*-test, stars indicate significance between control cell and individual IFITM (*P=<0.05).

Supplementary Figure 3. The P681R mutation does not confer IFITM resistance. A) cross symbol shows statistical significance by t-test between IFITM2 of D614G mutants compared to D614G, and statistical significance by t-test between IFITM2 of alpha mutants compared to the alpha of Figure 6. B) Hashtag symbol shows statistical significance by t-test between IFITM3 of the alpha mutants compared to the alpha of Figure 6. C) Representative immunoblots of cell lysates and purified supernatants of PLV production in 293T/17 cells. Virions were purified through a 20% sucrose cushion. D, E) Quantification of S2 over total S of cell lysates (D) and supernatant of PLVs produced in 293T/17 cells (E). F) A549-ACE2 cells stably expressing the individual IFITMs were infected with D614G P681R PLVs. PLV entry was quantified by Luciferase activity 48 hours after infection and infectivity normalized to control cells. **G)** A549-ACE2 cells stably expressing the individual IFITMs were infected with delta or delta R681P PLVs. PLV entry was quantified by Luciferase activity 48 hours after infection and infectivity normalized to control cells. **H)**. A549-ACE2s were pre-treated with 10μM E64d prior to transduction with D614G or D614G P681R PLVs and infection quantified by Luciferase activity 48h later. Data shown are mean ± SEM, n=3. Statistics were calculated in Prism using *t*-test, stars indicate significance between mock and individual IFITM or drug (*P=<0.05).

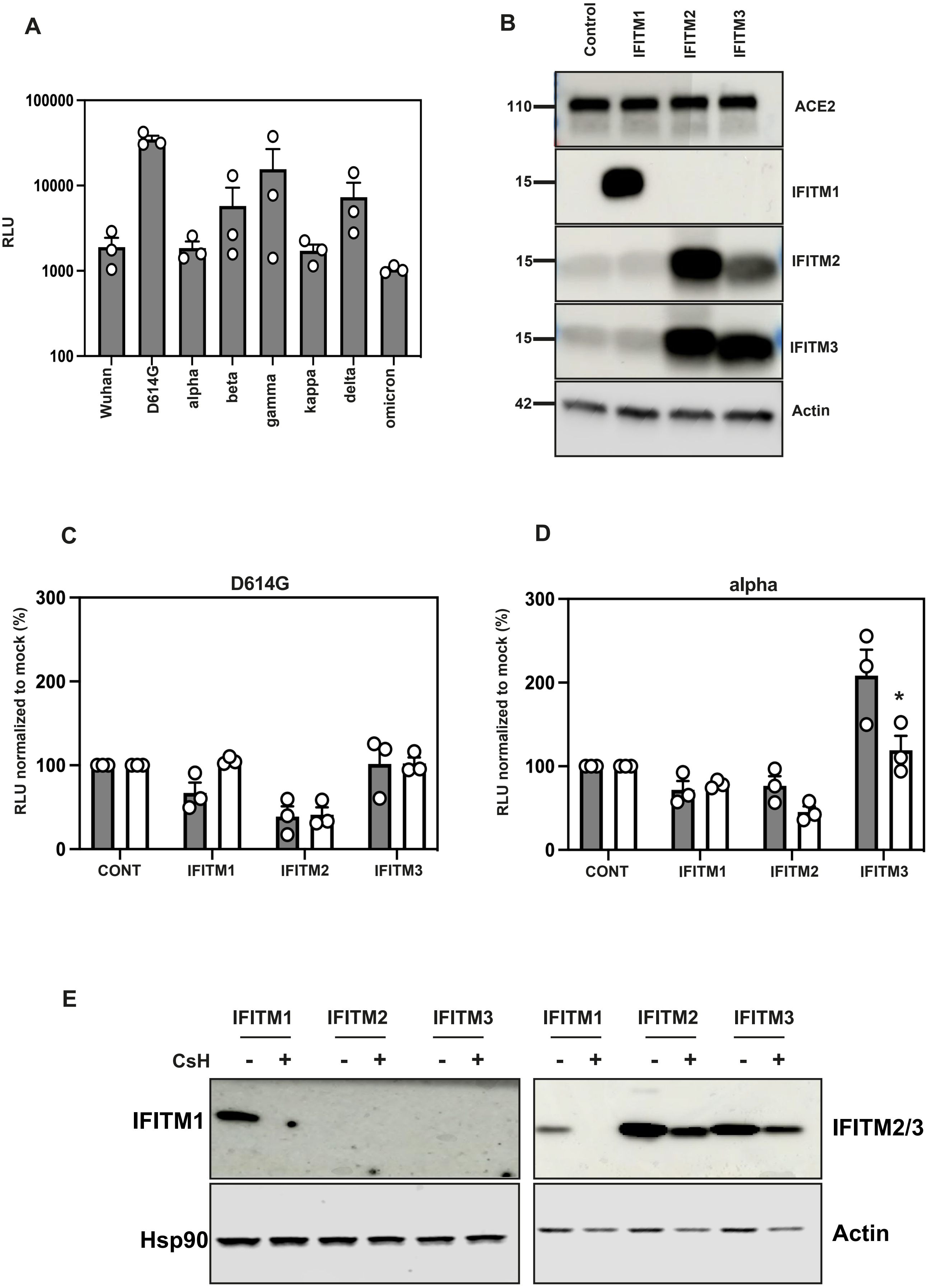

Supplementary figure 1

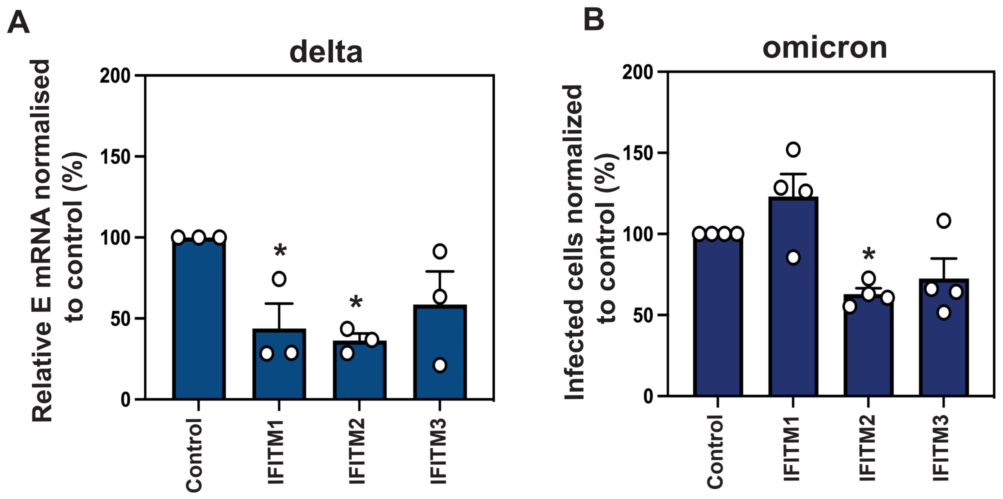

Supplementary figure 2

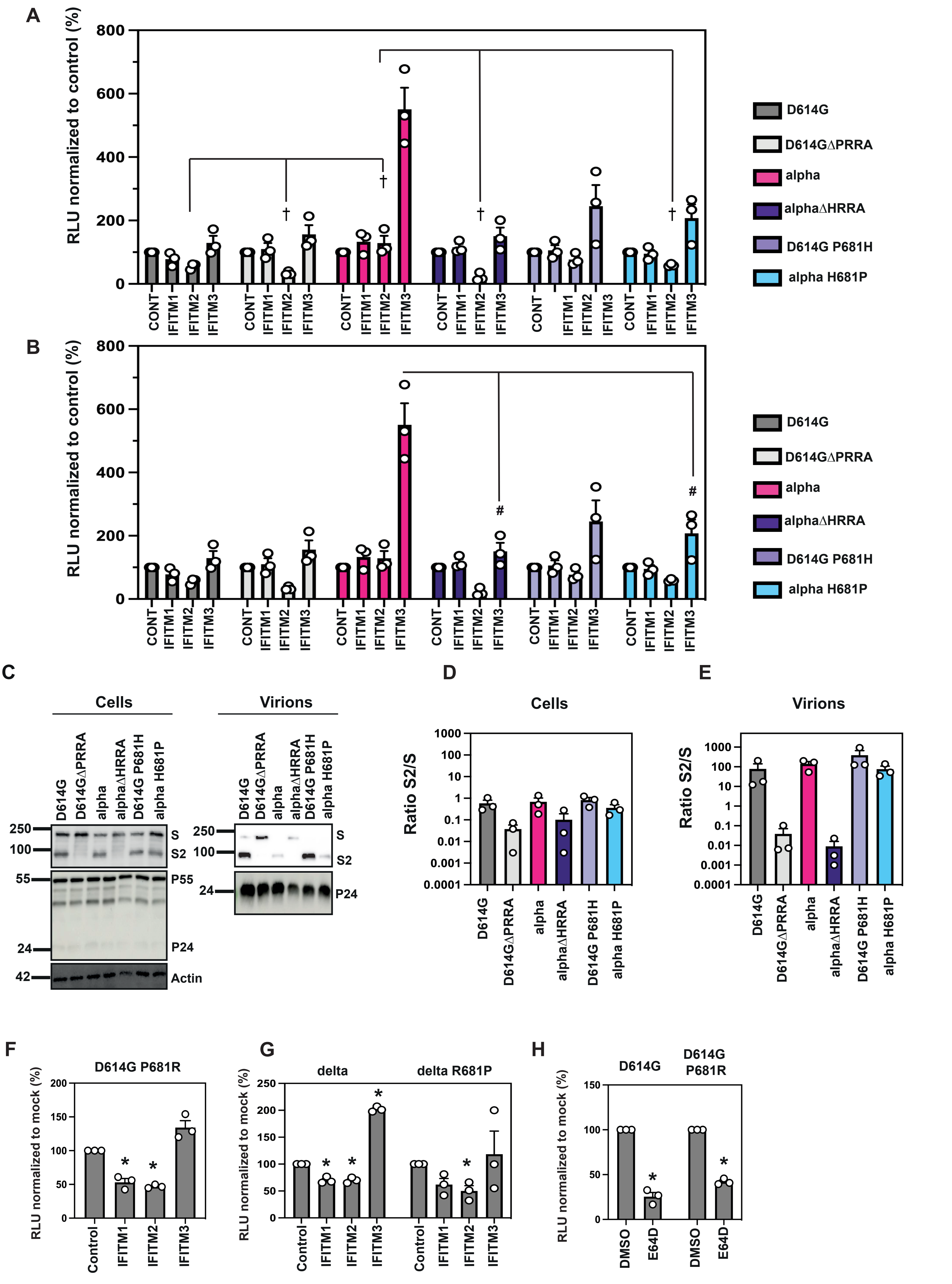

Supplementary figure 3
